# Evidence for dual pathways of *Tc1/mariner* domestication in *Drosophila*

**DOI:** 10.1186/s12915-026-02655-y

**Published:** 2026-06-16

**Authors:** Iuliia O. Guseva, Alexander P. Rezvykh, Elena S. Zelentsova, Dina A. Kulikova, Michael B. Evgen’ev, Sergei Y. Funikov

**Affiliations:** 1 Engelhardt Institute of Molecular Biology of Russian Academy of Sciences, Moscow, 119991 Russia; 2 Moscow Center for Advanced Studies, Kulakova Str. 20, Moscow, 123592 Russia; 3 Koltzov Institute of Developmental Biology, Russian Academy of Sciences, Moscow, 119334 Russia

**Keywords:** Transposable elements, *Tc1/mariner*, *Drosophila*, Genome evolution, Molecular domestication, Gene co-option, Transposase-derived genes

## Abstract

**Background:**

The domestication of transposable elements is a key source of evolutionary innovation, yet the pathways by which their functional modules are repurposed by the host remain poorly understood. The *Tc1/mariner* superfamily is a widespread group of DNA transposons, but the prevalence and patterns of their domestication are underexplored.

**Results:**

We performed a systematic genomic screen across 43 drosophilid species using stringent criteria for molecular domestication. This analysis identified five high-confidence, evolutionarily conserved genes derived from *Tc1/mariner* transposases*.* Phylogenetic and structural analyses suggest domestication via two distinct molecular pathways: co-option of the DNA-binding module and co-option of the catalytic domain. The DNA-binding module pathway includes *CG4570*, the previously known genes *cag* and *toy* (the latter fused with a homeodomain), and a lineage-restricted gene in the *Drosophila obscura* group that exhibits signatures of recent domestication. In contrast, the catalytic domain pathway is represented solely by *CG14478*. Structural modeling reveals that CG14478 protein preserves a canonical DDE endonuclease fold. Co-expression network analysis suggests potential cellular roles of these genes: *CG14478* is linked to RNA/chromatin-related processes, *CG4570* to cell cycle/chromosome functions, *cag* to ciliary and nuclear functions, and *toy* to neuronal development.

**Conclusions:**

This study establishes a stringent framework for identifying domesticated TEs, demonstrating that *Tc1/mariner* elements are co-opted via two distinct pathways: retention of either catalytic or DNA-binding modules. Our findings suggest that domestication is a dynamic continuum, ranging from recent, lineage-specific events to ancient, conserved genes, and underscore how genomic conflict with TEs can drive eukaryotic evolution and regulatory complexity.

**Supplementary Information:**

The online version contains supplementary material available at https://doi.org/10.1186/s12915-026-02655-y.

## Background

Once dismissed as “junk DNA,” transposable elements (TEs) are now recognized as pivotal drivers of evolution [1]. Beyond their inherent mobility, they serve as a major generative source of genetic novelty and regulatory innovation, thereby shaping adaptive genomic architectures and contributing directly to organismal fitness [2, 3]. The evolutionary process by which TEs transition from genomic parasites to functional host components is termed molecular domestication or exaptation [4–6]. This phenomenon occurs when host organisms co-opt TE-derived sequences that confer a selective benefit and are subsequently refined and maintained by natural selection.

The *Tc1/mariner* superfamily represents one of the most widespread and successful groups of DNA transposons found across diverse eukaryotic lineages, including animals, plants, fungi, and protists [7]. Phylogenetic analysis of transposase (TPase) sequences shows that the *Tc1/mariner* superfamily encompasses multiple distinct families. These include the major eukaryotic families *Tc1*, *mariner*, and *pogo*, along with their bacterial progenitor, the *IS630* family [8, 9]. Additionally, host-specific lineages have been identified, such as the *PlantMar* family in plants [7]. Structurally, *Tc1/mariner* transposons contain a central TPase gene flanked by terminal inverted repeats (TIRs), and integration generates short TA target site duplications (TSDs) [10, 11]. The TPase contains two essential functional domains: an N-terminal DNA-binding domain and a C-terminal catalytic domain containing the characteristic DDE/D motif (Asp-Asp-Glu/Asp) [10, 12]. The DNA-binding domain of *Tc1/mariner* TPases typically contains paired-box sequences (PAI and RED motifs) with helix-turn-helix structures that enable specific recognition of TIR sequences [13, 14]. The acidic triad DDE/D represents the catalytic core shared among all members of the *Tc1/mariner* superfamily of TPases and is crucial for the “cut-and-paste” transposition mechanism employed by these elements [15–17].

Domestication transforms parasitic TEs into functional host genes [4–6]. This process typically involves the separation of TPase coding sequences from their *cis*-acting TIRs, preventing further transposition while preserving protein-coding capacity. Once freed from the constraints of maintaining transposition machinery, domesticated TPases can evolve novel functions under host selection pressures. Several well-characterized examples illustrate the diverse functions acquired by domesticated TPases. *SETMAR*, found in hominids, represents a fusion between an *SET* histone methyltransferase gene and an *Hsmar1 mariner* TPase. Notably, the TPase-derived portion of SETMAR has lost its catalytic DDE triad and functions primarily as a DNA-binding module, directing the SET domain to specific genomic loci involved in DNA repair and transcriptional regulation [18–21]. The paired domain, a conserved DNA-binding module essential for acting as a master transcriptional regulator for key developmental genes (e.g., PAX family genes), originated from the DNA-binding domain of a *Tc1/mariner*-like TPase [22, 23]. CENP-B (centromere protein B) evolved from a *Tigger*-like element of the *pogo* family and plays crucial roles in centromere function and kinetochore assembly [24, 25]. The *pogo* family of *Tc1/mariner* TEs has been a remarkably prolific source of domesticated genes, with numerous independent domestication events occurring throughout tetrapod evolutionary history [26].

Although domestication events of the *Tc1/mariner* superfamily have been documented, it remains poorly understood whether they follow consistent evolutionary pathways, such as co-option of the catalytic module, the DNA-binding module, or both. With high-quality genomes and advanced genetic tools, drosophilid species, particularly those of the genus *Drosophila*, remain an indispensable model for investigating transposon domestication [27, 28]. The rich diversity of TEs has profoundly shaped genomic architecture and adaptation [29]. Recent advances in long-read sequencing and multi-omics now enable the detailed exploration of transposon-derived functions, such as domesticated nucleases, across evolutionary scales [30]. This positions *Drosophila* as a valuable system for investigating principles of transposon co-option in eukaryotes.

In this study, a systematic screen of 43 *Drosophila* genomes identified five genes unambiguously domesticated from the TPases of *Tc1/mariner* superfamily, while distinguishing them from numerous decaying pseudogenes and annotation artifacts that lack conserved synteny, expression, and selective constraint. Our results suggest that domestication proceeds via two distinct molecular pathways: one retaining the catalytic DDE domain (*CG14478*) and the other preserving only the DNA-binding domain (*CG4570*), alongside the previously known domesticated genes *cag* and *toy*. These four conserved genes exhibit signatures of ancient domestication, while a fifth lineage-restricted gene (*LOC26532114*) in the *D. obscura* group displays features of recent regulatory integration including moderate purifying selection, partial domain retention, and regulatory integration. Structural and co-expression analyses indicate that domesticated TPase derivatives have evolved specialized roles: CG14478 protein retains an intact DDE endonuclease fold and is associated with RNA/chromatin processes, while CG4570 protein, harboring only the ancestral DNA-binding domain, is linked to cell cycle and chromosome functions. These findings establish a dual-pathway framework for *Tc1/mariner* domestication and highlight the importance of multi-criteria approaches in distinguishing functional domestication events from genomic debris.

## Results

### Identification and phylogenetic distribution of domesticated genes derived from *Tc1/mariner* TPases in *Drosophila*

To systematically identify high-confidence domestication events of *Tc1/mariner* TPases in drosophilids, we performed a phylogenomic screen using a stringent set of criteria designed to distinguish stably integrated host genes from decaying transposon remnants [5, 31, 32]. First, its encoded protein should display significant sequence homology to a known TE protein (e.g., a TPase). Second, it must lack characteristics associated with transposition: the open reading frame (ORF) should not be flanked by transposon hallmarks such as TIRs and TSDs, and there should be no evidence of expansion, indicated by a low copy number. Third, it must possess intact coding capacity and evolve under selective constraints. Fourth, intact orthologs should be conserved across distantly related species, with preservation of local genomic context (microsynteny). Fifth, phylogenetic analysis should resolve the TE-derived orthologs as a distinct, monophyletic clade, separate from either their progenitor TE families or a representative set of *Tc1/mariner* TPase sequences. Sixth, there should be evidence of expression and a critical biological function in vivo.

Based on the first criteria, we systematically screened the 43 annotated drosophilid genomes, using tblastn, with queries representing *Tc1/mariner*-derived TPases from RepeatPeps (see Methods for details). This initial screen identified six genes harboring specific domains originating from *Tc1/mariner* TPases (Additional file 1: File S1). Upon applying our full domestication criteria, five of these were classified as domesticated genes. Four of these genes including *CG14478*, *CG4570*, *CG12346* (*cag*), and *CG11186* (*toy*) (FlyBase gene IDs) are widespread across the *Drosophila* genus. In contrast, the distribution of the fifth domesticated gene, *LOC26532114* (NCBI Gene ID), is restricted to the *D. obscura* species group. The sixth candidate and related sequences were later reclassified as truncated TE sequences or pseudogenes (see below).

Broader homology searches across eukaryotes indicate that proteins related to CG14478, CG4570, cag, and toy are distributed across a diverse array of metazoan and other eukaryotic lineages. In vertebrates, OrthoDB v12.2 [33] reveals that homologs of CG14478 and CG4570 are present in lineages extending up to and including Amphibia and Squamata, but are notably absent from Aves and all mammalian groups. In contrast, cag and toy exhibit a much wider phylogenetic distribution across vertebrates, including hominids. Human homologs of *cag* encompass members of the *TIGD* (Tigger transposable element derived) gene family and the well-characterized *CENP-B* gene [34], whereas toy homologs correspond to various members of the PAX (paired box) gene family [35]. Given their derivation from a repetitive TPase family, however, many of these similarities likely reflect independent, lineage-specific domestication events rather than strict orthology inherited from a single common ancestor.

To confirm that the identified genes are not active *Tc1/mariner* transposons, we examined all orthologs across the studied *Drosophila* genomes for the presence of TIRs and TSDs. As expected for elements that have lost transposition activity, all analyzed genes lacked the requisite TIRs and TSDs. Also, five candidate genes were shown to be present in *Drosophila* genomes at a low copy number.

Building upon this identification, we further investigate the molecular evolution of these *Tc1/mariner*-derived genes, distinguishing functional domestication events from pseudogenized remnants, and provide insights into their putative functions in vivo.

### Molecular evolution and expression of *CG14478*, a conserved DDE domain-retaining gene

During domestication, individual domains of TE proteins are often repurposed to serve new, host-beneficial roles [36–38]. This co-option is typically accompanied by the mutational degeneration of the other domains that are no longer required for transposition [39–43].

**Fig. 1:**
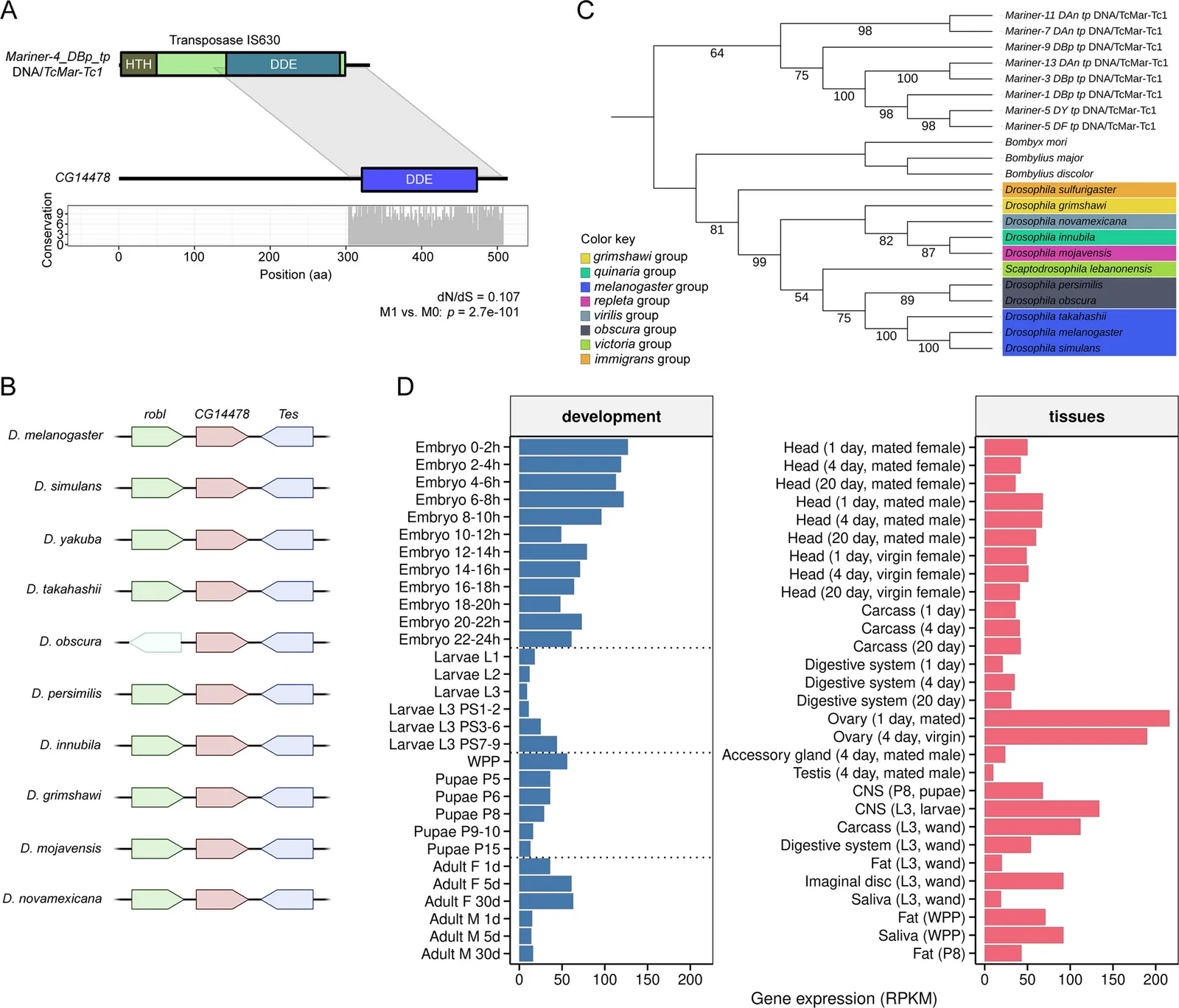
Molecular domestication of a *Tc1/mariner*-derived host gene *CG14478* in drosophilid species. **A** Schematic representation of pairwise alignment (gray regions) of the domesticated protein (bottom) with its ancestral TPase (top), identified as the best hit by tblastn analysis. Protein domains are indicated according to PFAM database. HTH—helix-turn-helix TPase DNA-binding domain; DDE—DDE superfamily endonuclease. A histogram panel displays the conservation of amino acid residues across the sequence alignment. A score of 11 indicates fully identical residues, while a score of 10 denotes residues with conserved physicochemical properties. Non-synonymous (dN) versus synonymous (dS) substitution rates (dN/dS) across orthologous sequences of the domesticated gene are indicated below. The reported dN/dS ratio is statistically supported by a likelihood ratio test (*p* < 0.05). **B** Maximum likelihood phylogenetic tree of the domesticated ortholog genes and a set of ancestral *Tc1/mariner* superfamily TPases. The ortholog of *CG14478* from *Bombyx mori* was used as an outgroup. Bootstrap support values after 100 replicates are shown. **C** Schematic representation of the genomic locus harboring the domesticated gene across multiple drosophilid species. Orthologs of domesticated genes are located in the center, demonstrating upstream and downstream microsynteny. The query gene and its flanking genes are assigned a different color, which is used to indicate ortholog counterparts in other species. Arrows indicate transcription direction. The query gene and its flanking sequences are highlighted in a distinct color to indicate orthology with *D. melanogaster* across species. Genes shaded in a lighter tone are non-orthologous. Arrows denote the direction of transcription. **D** Normalized expression (RPKM) of the *CG14478* gene from developmental stage- and tissue-specific RNA-seq datasets

Based on tblastn alignment, only the C-terminal region of the *Tc1/mariner* TPase showed homology to *CG14478* (score 146, *e* value 3e − 44) (Fig. 1A; Additional file 1: File S1). Notably, this homologous region encompassed the entire DDE catalytic domain within the TE-derived gene protein (Fig. 1A). Conserved Domain Database (CDD) [44] searches confirm that the only detectable structured domain in CG14478 is the C-terminal DDE endonuclease domain of TPase origin; the N-terminal region shows no homology to other known domains. A blast analysis of the translated N-terminal domain of CG14478 against TEs of invertebrates from RepBase v27.01 and annotated *Drosophila* gene sequences revealed homology exclusively to *CG14478* itself and its orthologs, with no significant matches (*e* value < 0.05) to other protein-coding genes or known TEs. This suggests that the N-terminal region of the protein encoded by the *CG14478* gene likely evolved from the same ancestral transposon, but has diverged beyond recognition in the course of evolution.

After proliferating in a genome, TPase genes are thought to evolve under neutral selection [45]. In contrast, if domesticated TPase genes serve a genuine cellular function for the host, their evolution should be shaped by either purifying selection to preserve their function or positive selection resulted in adaptation. To evaluate selective pressures on *CG14478*, we compared the non-synonymous to synonymous substitution ratio (dN/dS) using orthologs from the *Drosophila* genus. This analysis indicated that *CG14478* is subject to purifying selection (dN/dS = 0.107) (Fig. 1A). Likelihood ratio tests (LRT) confirmed that the estimated dN/dS ratios are statistically significant. For *CG14478*, the model incorporating purifying selection and neutral sites (M1) fit the data significantly better than the null model (M0) (*p* = 2.7e − 101).

To confirm that the candidate genes have not undergone transposition, we assessed the preservation of microsynteny across the studied *Drosophila* genomes. Analysis of the *Drosophila* genus revealed a broadly conserved microsyntenic arrangement flanking the *CG14478* gene (Fig. 1B). This conservation was observed in both upstream and downstream genomic regions across virtually all examined species. A notable exception was identified in *Drosophila obscura*, where synteny was maintained only in the downstream region of the gene (Fig. 1B).

Phylogenetic analysis demonstrated that *CG14478* orthologs form a monophyletic clade distinct from the clade of reference *Tc1/mariner* TPases, congruent with the *Drosophila* species phylogeny (Fig. 1C) [46, 47]. Together with high sequence conservation and microsynteny, this phylogeny indicates strong functional constraints on the CG14478 protein, providing additional evidence for its domestication.

To assess the expression pattern of *CG14478*, we analyzed publicly available modEncode RNA-seq data from *D. melanogaster* [48]. The developmental expression profile demonstrates that *CG14478* is expressed throughout embryogenesis and is notably abundant in the adult females (RPKM > 50) (Fig. 1D). Analysis of expression patterns across different tissues indicated that *CG14478* shows maximal expression in ovarian tissue (RPKM > 150), with lower but detectable expression in the head, central nervous system (CNS), third-instar larval imaginal discs, as well as in fat body and salivary glands at the prepupal stage (RPKM > 50) (Fig. 1D).

Collectively, these observations are consistent with *CG14478* being a domesticated derivative of a *Tc1/mariner* TPase. Its origin is marked by the co-option and preservation of the ancestral catalytic DDE domain, while the accompanying HTH domain has undergone extreme divergence. Furthermore, its ubiquitous but regulated expression, with notable enrichment in ovarian tissue, suggests a conserved cellular role that emerged following domestication.

### Molecular evolution and expression of *CG4570*, a *Tc1/mariner* DNA-binding domain-derived gene

**Fig. 2:**
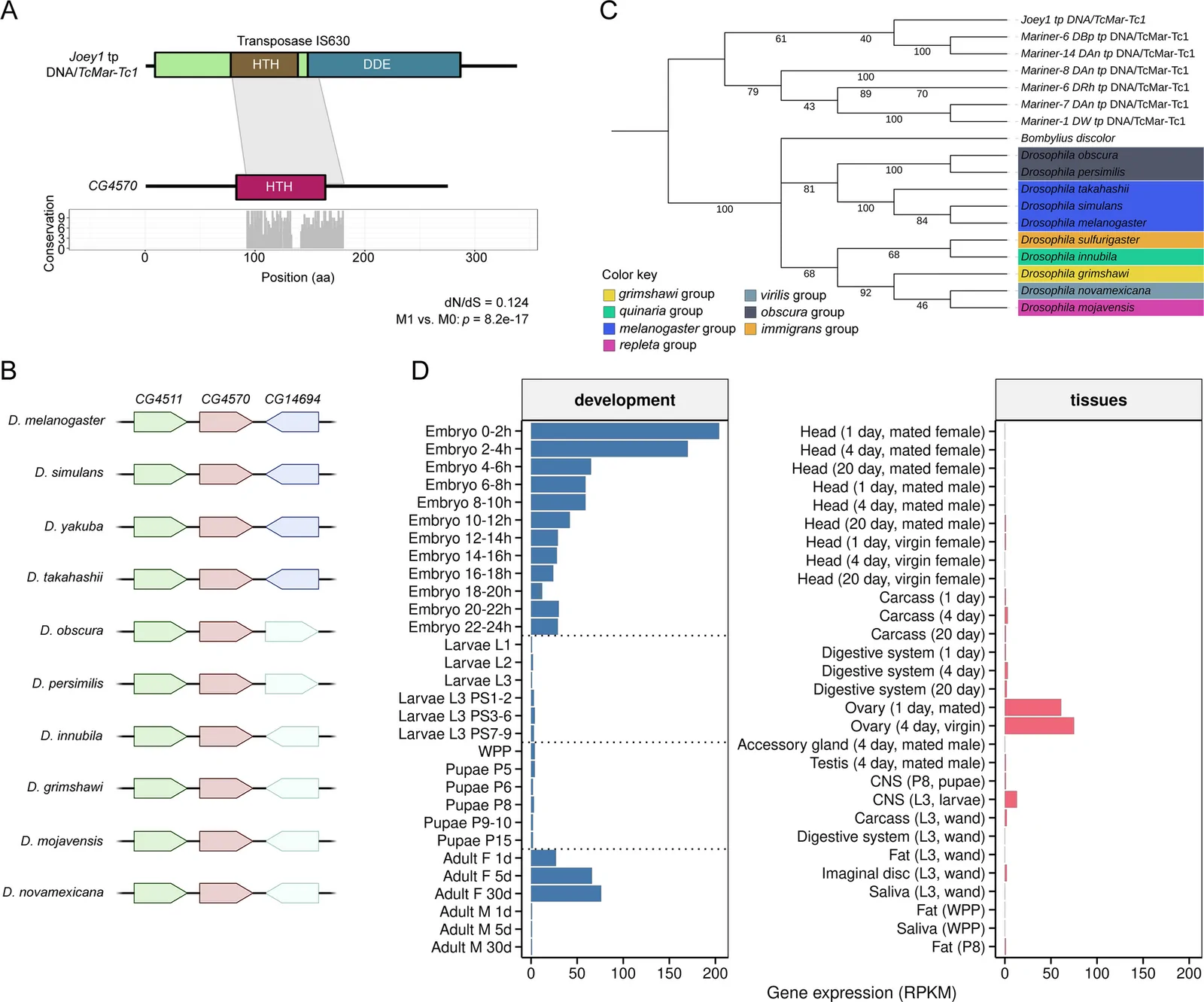
Molecular domestication of a *Tc1/mariner*-derived gene *CG4570* in *Drosophila*. **A** Scheme of a pairwise alignment (gray regions) between the domesticated protein (bottom) and its ancestral TPase (top), identified via tblastn. Domains are annotated per the PFAM database. HTH—helix-turn-helix TPase DNA-binding domain; DDE—DDE superfamily endonuclease. A histogram shows amino acid conservation across the alignment (score: 11 = identical, 10 = conserved properties). The dN/dS ratio of orthologs of the domesticated gene is indicated on the right. The reported dN/dS ratio is statistically supported by a likelihood ratio test (*p* < 0.05). **B** Maximum likelihood phylogeny of the domesticated orthologs and a set of ancestral *Tc1/mariner* superfamily TPases. The ortholog of *CG4570* from *Bombylius discolor* was used as an outgroup. Bootstrap support values after 100 replicates are shown. **C** Genomic microsynteny of the locus containing the domesticated gene across *Drosophila* species. The orthologous region from *D. melanogaster*, including the query gene and its surrounding sequences, is consistently highlighted in a unique color across all species. Non-orthologous genes are shown in a faded color. Transcription direction is indicated by arrows. **D** Normalized expression (RPKM) of *CG4570* from developmental stage- and tissue-specific RNA-seq datasets

In contrast to *CG14478*, sequence homology analysis indicates that only the N-terminal region of the *Tc1/mariner* TPase, encompassing the HTH domain, is homologous to *CG4570* (tblastn score 46.6, *e* value 2e − 10) (Fig. 2A, Additional file 1: File S1). Furthermore, the protein encoded by *CG4570* (225 aa) is significantly shorter than its ancestral TPases (338 aa), consistent with the co-option of solely the DNA-binding domain. CDD searches indicate that the only conserved domain in the truncated CG4570 protein is the N-terminal HTH DNA-binding domain derived from the ancestral TPase.

Similar to *CG14478*, the evaluation of selective pressures via dN/dS analysis indicated that *CG4570* is under strong purifying selection (dN/dS = 0.124) (Fig. 2A). LRT for *CG4570* showed strong statistical support for purifying selection (M1 vs M0: *p* = 8.2e − 17). Analysis of microsynteny conservation revealed preserved gene order upstream and downstream of the *CG4570* locus across the *D. melanogaster* species group. Additionally, upstream microsynteny was maintained in more distantly related *Drosophila* species (Fig. 2B). These findings indicate a stable genomic position for *CG4570* and a lack of recent transposition events. Phylogenetic reconstruction revealed that *CG4570* orthologs form a monophyletic clade separate from reference *Tc1/mariner* TPase sequences (Fig. 2C).

Gene expression analysis revealed that *CG4570* transcripts are detectable throughout embryogenesis, with peak expression occurring in 0–2-h embryos (RPKM ~ 200) followed by a sharp decline in later embryonic stages (RPKM ~ 25) (Fig. 2D). Beyond embryogenesis, expression is predominantly observed in adult females (RPKM > 50), consistent with active transcription in ovarian tissue (Fig. 2D). The high transcript abundance in very early embryos is likely attributable to maternally deposited mRNA derived from ovarian expression, suggesting that *CG4570* may function as a maternal contribution to early embryonic processes. A low level of expression is also observed in the central nervous system (CNS) of third-instar larvae (RPKM = 16) (Fig. 2D).

In summary, *CG4570* represents a derived, domesticated gene originating from the *Tc1/mariner* TPase lineage. Through a process of domain co-option, only the ancestral N-terminal HTH DNA-binding domain has been retained, resulting in a significantly truncated, stable protein-coding gene. The gene shows a spatially and temporally restricted expression pattern, predominantly during early embryogenesis and in adult female tissues. This suggests a specialized, likely regulatory role in *Drosophila* development, potentially related to ovarian function or/and early embryonic processes.

### Re-evaluation of domesticated *Tc1/mariner*-derived genes: *cag* and *toy*

The genes *cag* and *toy* (*twin of eyeless*) are documented examples of *Tc1/mariner* TPase domestication [23, 25, 49, 50]. Following established criteria for domesticated TEs, we re-evaluated their molecular evolution and expression patterns.

The *cag* gene originates from the *pogo* family of *Tc1/mariner* transposons and has inherited a CENP-B-type DNA-binding domain, an evolutionarily modified HTH fold (tblastn score 81.3, *e* value 4e − 22) (Additional file 2: Fig. S1A; Additional file 1: File S1). Homology between *toy* and TPase of *Tc1/mariner* elements is confined to the HTH domain (tblastn score 44.7, *e* value 3e − 09) (Additional file 3: Fig. S2A, Additional file 1: File S1). The homeodomain of PAX proteins likely originated from ancestral homeobox domain capture, resulting in a stable fusion [23, 51].

Selective pressure analysis via dN/dS computation revealed that both genes are under purifying selection, indicative of functional constraint. The *cag* gene exhibits a dN/dS ratio of 0.195, while *toy* shows an even stronger selective constraint with a dN/dS ratio of 0.049 (Additional file 2: Fig. S1A; Additional file 3: Fig. S2A). Statistical support for purifying selection was confirmed by likelihood ratio tests for both *cag* (M1 vs M0: *p* = 7.1e − 18) and *toy*, for which the model assuming uniform purifying selection across all sites (M0) provided the best fit.

To contextualize these selection pressures, we compared our observed dN/dS ratios against a published genome-wide baseline. A comprehensive analysis of conserved orthologs across 12 *Drosophila* species (flyDIVaS) established that dN/dS values for conserved single-copy genes under long-term purifying selection typically range from 0.02 to 0.15 [52]. The ratios we observed for *CG14478* (dN/dS = 0.107), *CG4570* (dN/dS = 0.124), and *toy* (dN/dS = 0.049) fall directly within this interval, and *cag* (dN/dS = 0.195) is only marginally higher. This concordance indicates that the selective constraints operating on these transposon-derived genes are effectively indistinguishable from those governing a wide array of evolutionarily conserved host genes, strongly supporting their functional significance.

Microsynteny surrounding both loci was conserved across the *D. melanogaster* species group, with at least downstream synteny maintained in more distant species (Additional file 2: Fig. S1B; Additional file 3: Fig. S2B). However, the local gene order of *cag* was disrupted in *Drosophila innubila* and the *Drosophila virilis* species group, likely due to chromosomal inversions (Additional file 2: Fig. S1B). Phylogenetic reconstruction confirmed that orthologs of *cag* and *toy* form monophyletic clades distinct from reference *Tc1/mariner* TPase clades (Additional file 2: Fig. S1C; Additional file 3: Fig. S2C), consistent with their derivation and subsequent evolution as host genes.

Expression analysis of modEncode RNA-seq data revealed distinct profiles for each gene. Gene *cag* exhibits consistently low expression across all developmental stages and tissues, with its highest levels detected in 10–12-h embryos and testis (Additional file 2: Fig. S1D). Similarly, *toy* shows low, ubiquitous expression during most of development, though it is completely absent in 0–2-h embryos and in 30-day-old females (Additional file 3: Fig. S2D). Tissue-specific profiling indicated that *toy* expression is largely restricted to heads and the larval CNS (Additional file 3: Fig. S2D).

Collectively, these findings reinforce the classification of *cag* and *toy* as domesticated derivatives of *Tc1/mariner* TPases.

### Mild purifying selection and regulatory integration in a recently domesticated TPase

Our analysis identified *LOC26532114* in *D. pseudoobscura* as a potential *Tc1/mariner*-derived gene. However, its features suggest a more complex lineage-specific evolutionary history compared to widely conserved genes described in previous sections.

**Fig. 3:**
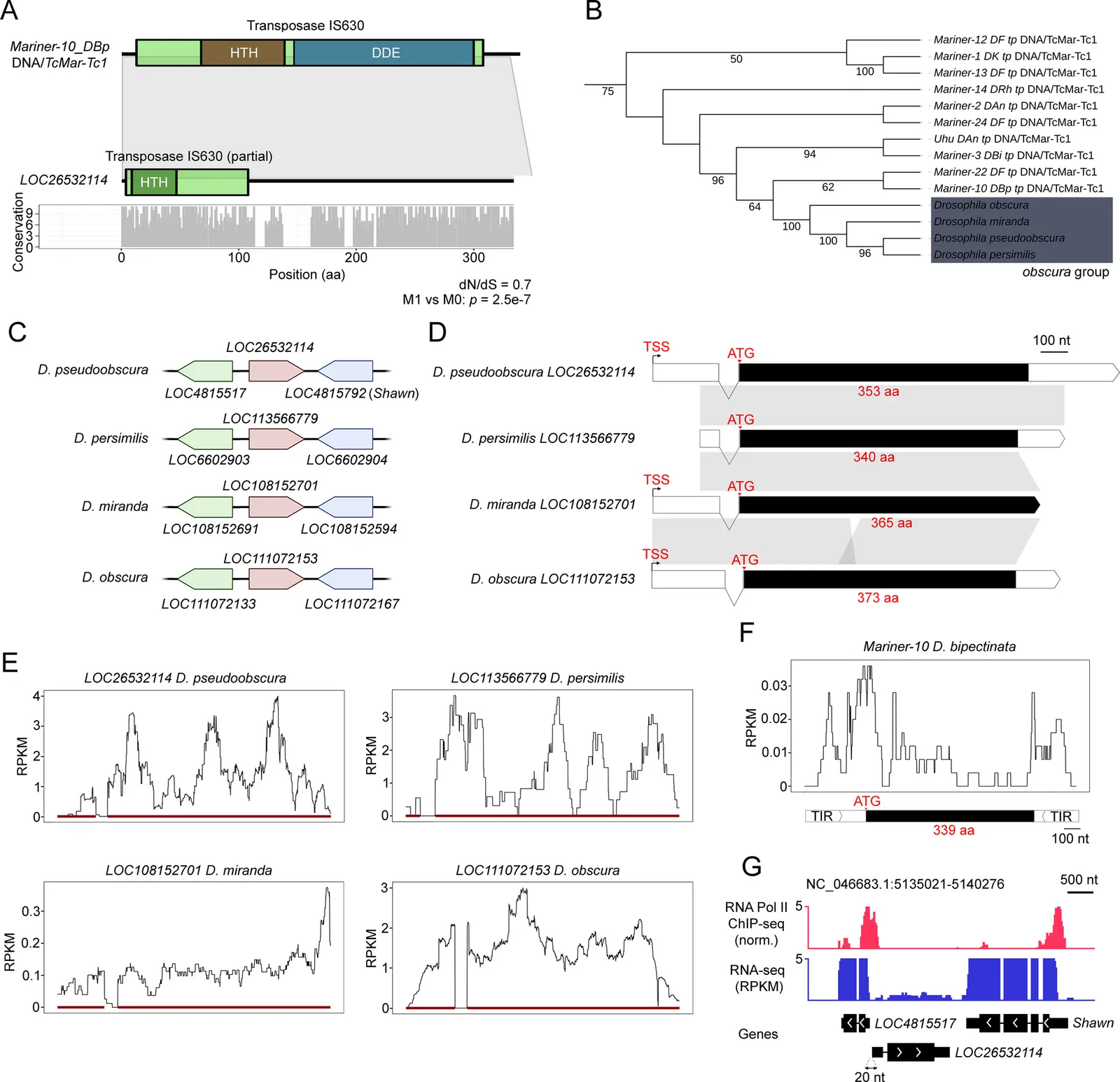
Molecular domestication of a *LOC26532114* gene derived from a *Tc1/mariner* TPase in *D. obscura* species group. **A** Pairwise alignment (gray) between the domesticated protein (bottom) and its ancestral TPase (top), identified by tblastn. Domains are annotated according to PFAM. HTH—helix-turn-helix TPase DNA-binding domain; DDE—DDE superfamily endonuclease. A histogram shows amino acid conservation across the alignment (score: 11 = identical, 10 = conserved properties). The dN/dS ratio for orthologs of the domesticated gene is displayed at the right. The reported dN/dS ratio is statistically supported by a likelihood ratio test (*p* < 0.05). **B** Maximum likelihood phylogeny of the domesticated orthologs and a selection of ancestral *Tc1/mariner* superfamily TPases. Bootstrap values after 100 replicates are shown. **C** Genomic microsynteny of the locus containing the domesticated gene across *D. obscura* species. The orthologous region from *D. pseudoobscura*, including the query gene and its flanking sequences, is uniformly highlighted in a distinct color in all species. Arrows indicate transcriptional direction. **D** Comparative gene organization of *LOC26532114* and its orthologs. White and black regions on the scheme denote untranslated and coding regions, respectively. The gray area between genes indicates homologous regions defined using nucleotide blastn basing on the percentage of identity not less than 70. The rightwards corner arrow at the beginning of genes indicates the transcriptional start site. The 3′ and 5′-untranslated regions are given according to their annotations, except for *D. obscura* (see text). **E** RPKM-normalized expression in whole female bodies and sequencing coverage profiles across gene bodies. **F** RPKM-normalized expression in whole female bodies and sequencing coverage profiles across *Mariner-10* in *D. bipectinata*. **G** Locus profile of *LOC26532114* in *D. pseudoobscura* with upstream and downstream flanking genes depicting enrichment of RNA Pol II in the TSS and 5′UTRs of genes

Sequence homology analysis via tblastn revealed that *LOC26532114* exhibits strong similarity to ancestral *Tc1/mariner* TPases (score 249, *e* value 3e − 85), with the alignment spanning nearly the entire length of the TPase (Fig. 3A, Additional file 1: File S1). However, a detailed protein domain search indicated a pattern of partial domain retention. While the N-terminal HTH domain remains intact, the C-terminal catalytic region shows only a partial preservation of the IS630 TPase domain, with no detectable conservation of the canonical DDE domain (Fig. 3A).

As noted before, phylogenetic distribution analysis according to OrthoDB v12.2 shows that *LOC26532114* is not widely conserved in *Drosophila*. Orthologs are found only in the *D. obscura* species group including *D. pseudoobscura*, *Drosophila persimilis*, *Drosophila miranda*, and *Drosophila obscura*.

The dN/dS ratio of 0.7 for *LOC26532114* indicates moderate/mild purifying selection, placing it between neutral evolution (≈1) and the strong constraint seen in genes like *CG14478* (dN/dS = 0.107, M1 vs M0: *p* = 2.5e − 7) (Figs. 3A and 1A).

Phylogenetic reconstruction further corroborated its domesticated status, placing its orthologs in a monophyletic clade distinct from clades of *Tc1/mariner* TPases (Fig. 3B). Microsynteny analysis confirmed a stable genomic context for *LOC26532114* within the related species, with conserved gene order both upstream and downstream of the locus (Fig. 3C).

To better characterize the domestication of *LOC26532114* and its orthologs, we compared their exon–intron structures. This analysis revealed a conserved intron in the 5′ untranslated region (UTR) upstream of the translation initiation codon (Fig. 3D). Note, analysis of the *LOC26532114* ortholog in *D. obscura* (*LOC111072153*) identified a potential misannotation of its ORF. The annotated ORF was substantially longer than those in related species. A Kozak sequence prediction analysis [53] indicated a higher probability of translation initiation at a downstream ATG (position 274; score 0.77) rather than the annotated upstream site (position 151; score 0.65) (Additional file 4: Fig. S3). This downstream start aligns with other orthologs and was used for all conducted analyses.

Expression analysis of *LOC26532114* in adult females of four *D. obscura* group species revealed modest, yet consistent, expression levels across three of the four species examined (Fig. 3E). *D. miranda* represents a marked exception, where transcript abundance was approximately one order of magnitude lower than in related species (Fig. 3E).

Inspection of RNA-seq read coverage confirmed the splicing of the conserved 5′ UTR intron, as reads did not align to this region (Fig. 3E). Notably, the ancestral *Mariner-10* transposon from *D. bipectinata* lacks this intron (Fig. 3F), suggesting its acquisition in the domesticated lineage as part of the integration into host gene regulatory architecture.

Importantly, the transcriptional start site (TSS) of the upstream gene *LOC4815517* (orthologous to *D. melanogaster CG14210*) is located only 20 base pairs from the TSS of *LOC26532114* in *D. pseudoobscura*, a spacing that is conserved in its orthologs (Fig. 3G). ChIP-seq data demonstrate that RNA polymerase II occupancy spans the genomic region encompassing both genes (Fig. 3G). This minimal intergenic distance suggests that the two genes likely utilize a shared bidirectional promoter, consistent with the evolutionary co-option of this regulatory architecture for the transcription of the domesticated TPase.

This mode of regulatory acquisition is not unique to recently domesticated genes. Among the ancient conserved genes identified in this study, *cag* similarly shares a bidirectional promoter with the adjacent gene *CG12942/dumpless1*, and their expression is significantly correlated (Spearman *R* = 0.624, *p* = 1.27e − 7). In contrast, the other conserved domesticated genes (*CG14478*, *CG4570*, *toy*) lack evidence of promoter sharing or coordinated expression with neighboring genes.

In summary, *LOC26532114* represents a candidate domesticated gene derived from a *Tc1/mariner* TPase, with its occurrence restricted to the *D. obscura* species group. Its evolutionary trajectory is marked by the loss of the catalytic DDE domain, mild selective constraint, and the acquisition of a stable intron in its regulatory region. These features collectively portray *LOC26532114* as a potential example of a recent, lineage-specific domestication event, where the gene may be in the process of functional refinement.

### Distinguishing decaying pseudogenes from bona fide domesticated genes

In our systematic survey for domesticated TPases, we identified a set of putative *Tc1/mariner*-derived coding sequences in several phylogenetically distant *Drosophila* species. These sequences include single-copy candidates such as *LOC122624997* in *Drosophila teissieri*, *LOC123257900* in *Drosophila ananassae*, *LOC117781342* in *Drosophila innubila*, and *LOC127565796* in *Drosophila albomicans*, as well as candidates present in two copies, including *LOC123037293* and *LOC123037367* in *Drosophila rhopaloa*, *LOC122322526* and *LOC122322551* in *Drosophila grimshawi*, and *LOC128263834* and *LOC128263967* in *Drosophila gunungcola* (Additional file 1: File S1).

Although these sequences exhibit pronounced amino acid similarity to canonical *Tc1/mariner* TPases and lack the TIRs and TSDs characteristic of active TEs, several lines of evidence argue against their classification as domesticated host genes. First, microsynteny analysis revealed no conservation of genomic context either upstream or downstream of these loci across species (Additional file 5: Fig. S4A). Second, the gene structures of these candidates are highly divergent from one another, precluding their assignment as orthologs (Additional file 5: Fig. S4B). Third, phylogenetic reconstruction failed to resolve these sequences into a monophyletic clade distinct from reference *Tc1/mariner* lineages. Instead, they tended to cluster within or adjacent to branches containing potentially active transposons (Additional file 5: Fig. S4C). Analysis of available RNA-seq data reveals misalignment of reads relative to annotated exon–intron structures, concurrent with markedly low or undetectable expression levels, as observed in *Drosophila ananassae* (Additional file 5: Fig. S4D).

These observations suggest that these loci more likely represent truncated TE copies or decaying pseudogenes, rather than genuine, functionally domesticated genes. Their sporadic distribution, lack of conserved synteny, and absence of expression highlight the importance of applying stringent, multi-criteria assessments to distinguish between true molecular domestication events and the abundant genomic debris left by transposon activity.

### Structural and functional conservation of the DDE motif in CG14478 protein

The DDE/D motif constitutes a conserved catalytic core within TPase, characterized by two invariant aspartic acid (D) residues and a third catalytic residue which is either aspartate (D) or glutamate (E) [8, 10]. The distance between the first two “D” amino acids is variable, while the distance between the second “D” and the third “D/E” is highly conserved [54–56].

**Fig. 4:**
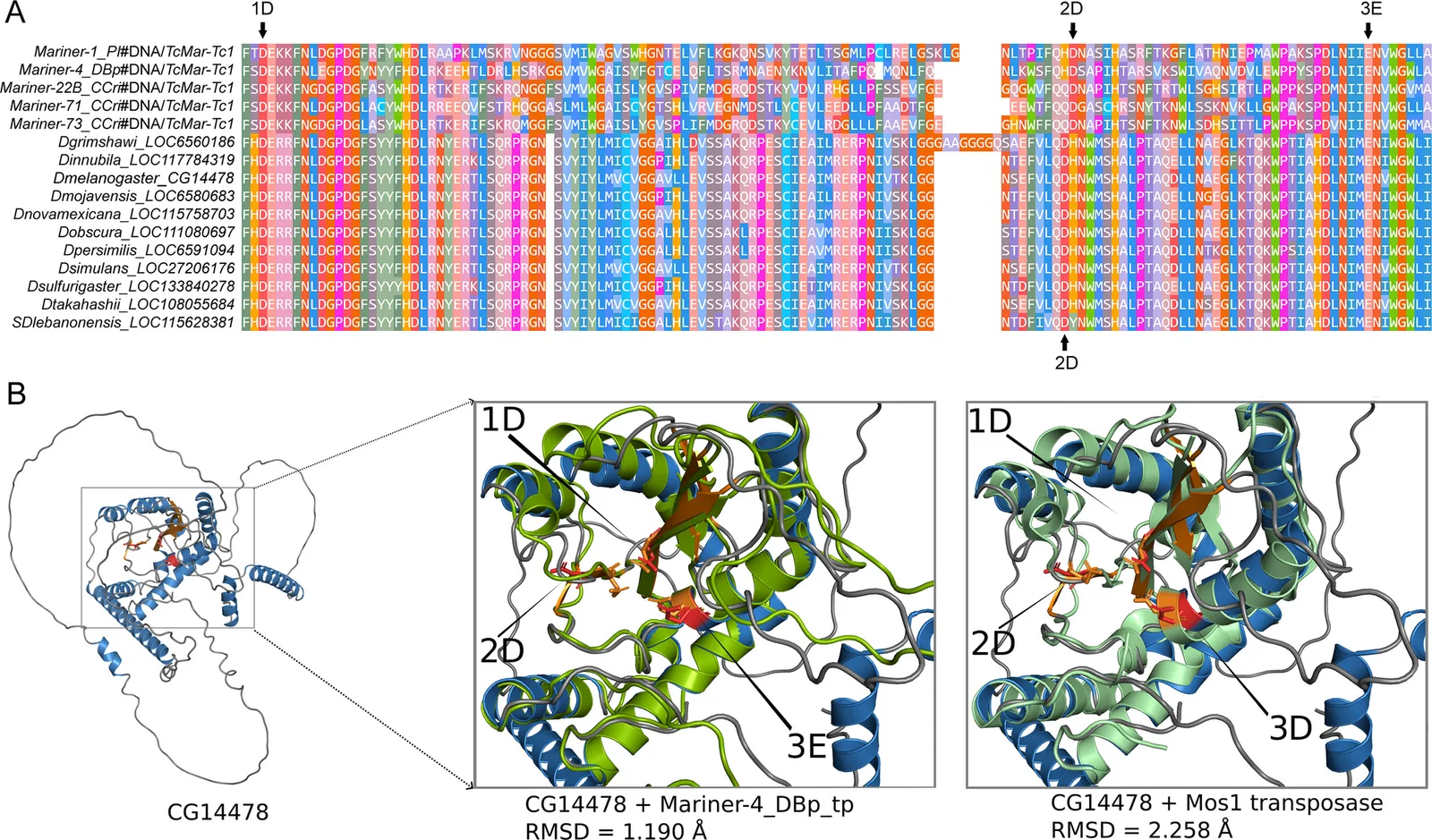
Structural analysis of the DDE catalytic motif in CG14478. **A** Multiple sequence alignment of CG14478 orthologs and ancestral TPases, focused on regions containing the conserved DDE catalytic domains. The three essential catalytic residues (DDE) are marked with arrows for *Tc1/mariner*-derived CG14478 orthologs and ancestral TPases. **B** Left: the predicted tertiary structure of CG14478 generated by AlphaFold2. Alpha-helices are rendered in blue, and beta-sheets are in orange. The catalytic aspartate and glutamate residues forming the conserved DDE endonuclease motif are highlighted in red. Center: spatial alignment of the DDE domains from the predicted AlphaFold model for Mariner-4_DBp_tp TPase (in light green) and CG14478. The conserved catalytic triads for CG1477 and for Mariner-4_DBp_tp are shown in red and orange, respectively. Right: spatial alignment of the DDE endonuclease domains, focused on the superposition of the catalytic domain of the Mos1 TPase crystal structure (pistachio) onto the predicted tertiary structure of CG14478. Catalytic DDE/D residues are indicated in red for CG14478 (3E) and orange for Mos1 (3D). All three structural representations are presented from an identical viewpoint. RMSD (root mean square deviation) values are shown for each alignment

The only identified gene of putative *Tc1/mariner* TPase origin that retains the DDE/D domain is *CG14478*. To assess its functional potential, we performed a multiple sequence alignment with both its orthologs and ancestral *Tc1/mariner* TPases. We found that CG14478 protein retains an intact DDE motif across all examined orthologs (Fig. 4A). However, a notable deviation is observed in the spacing between the second D (D2) and the third E (E3) residues. Where ancestral TPases maintain a characteristic 35-amino-acid interval, CG14478 exhibits a 36-amino-acid span, representing a one-residue insertion within this otherwise conserved catalytic architecture (Fig. 4A). This preserved yet slightly altered spacing suggests CG14478 may have adapted the ancestral catalytic scaffold while maintaining functional potential.

To define the structural architecture of the CG14478 protein, we analyzed its AlphaFold-predicted 3D model (see Methods for details). Analysis of the CG14478 protein sequence (512 residues) via the CDD identified a canonical DDE endonuclease domain spanning residues 322–473 (Fig. 1A). This localization was subsequently validated by 3D structural modeling, which confirmed the presence of the conserved DDE structural fold within this region (Fig. 4B). The predicted local distance difference test (pLDDT) confidence scores for DDE domain are ≥ 70, indicating a model of acceptable reliability [57]. In contrast, the N-terminal segment (residues 1–321) exhibits features consistent with intrinsic structural disorder (Fig. 4B). Predictive structural analysis reveals few isolated α-helical elements embedded within a presumably flexible sequence (pLDDT < 50). The structural absence of a canonical globular fold, coupled with a sparse and fragmented secondary structure profile across this extensive sequence, is indicative of an intrinsically disordered region (IDR). To computationally validate this assessment, we performed disorder prediction using AIUPred, an algorithm that integrates biophysical energy estimations with deep learning-based inferences [58]. The analysis robustly identified residues 107–261 of CG14478 as possessing a high propensity for disorder (score ~ 1) (Additional file 6: Fig. S5), thereby providing strong in silico support for the classification of the N-terminal segment as an IDR.

The conserved DDE/D catalytic triad of TPases is known to form a characteristic “pocket” which coordinates divalent metal ions essential for the transposition reaction [10]. To determine whether the DDE domain of CG14478 retains this canonical structural architecture, we performed a structural comparison between the AlphaFold-predicted models of CG14478 and the ancestral Mariner-4 TPase from *D. bipectinata*, previously identified via blast search. Both structures exhibited a similar pocket-like topology (Fig. 4B). Spatial pairwise alignment of DDE domains using PyMOL [59] yielded a high-quality match (alignment score: 850.4; RMSD: 1.190 Å), with canonical α-helices and β-strands superimposing accurately. Strikingly, a high degree of spatial conservation was observed for two catalytic residues (designated D1 and E3). Specifically, D146 in Mariner-4 and D327 in CG14478, along with E268 in Mariner-4 and E449 in CG14478, occupied nearly superimposable positions within the active site (Fig. 4B). A third conserved residue (D2), corresponding to D231 in Mariner-4 and D413 in CG14478, maintained a comparable backbone position. However, a significant conformational divergence was noted—the side-chain carboxyl group of D413 in CG14478 was rotated approximately 180° relative to its counterpart, orienting it away from the catalytic center of Mariner-4 (Fig. 4B).

Additionally, the CG14478 was compared to the crystallographically resolved structure of catalytic domain of the *mariner* Mos1 TPase. Analysis of the Mos1 and CG14478 catalytic domain structures confirmed a conserved overall fold and maintained spatial arrangement of the catalytic triad. This conservation persists despite a difference in the identity of the third residue within the DDE/D triad between Mos1 (3D) and CG14478 (3E) (Fig. 4B). However, the overall structural alignment quality was lower (alignment score: 659.8 588.4; RMSD: 2.258 Å). This decrease is likely attributable to the greater evolutionary divergence of the Mos1 TPase lineage from that of Mariner-4 and, consequently, from CG14478 [60]. The slightly reduced alignment quality with Mos1 also underscores the structural nuance likely introduced by the D2–E3 spacing shift in CG14478.

In summary, CG14478 protein retains the canonical DDE structural fold but carries a one-residue insertion between the second and third catalytic residues. This minor spacing alteration, coupled with a reoriented D2 side chain, suggests a structural divergence that may reflect functional adaptation or regulatory modulation, while still supporting the hypothesis that *CG14478* encodes an active endonuclease derived from a *Tc1/mariner* element.

### Co-expression network analysis suggests potential cellular roles for *Tc1/mariner*-derived genes

To infer the potential cellular roles of the domesticated *Tc1/mariner*-derived genes identified in this study, we performed a gene co-expression network analysis for *CG14478*, *CG4570*, *cag*, and *toy* using developmental and tissue-specific expression data from *D. melanogaster*. Genes with a Spearman’s correlation coefficient > 0.7 (*p* < 0.05) relative to each query gene were considered co-expressed (see Methods for details). This analysis yielded independent co-expression networks for *CG14478* (790 genes), *CG4570* (888 genes), *cag* (3182 genes), and *toy* (398 genes). Subsequent Gene Ontology (GO) enrichment analysis of these networks provided functional predictions consistent with the known or inferred properties of these domesticated proteins.

**Fig. 5:**
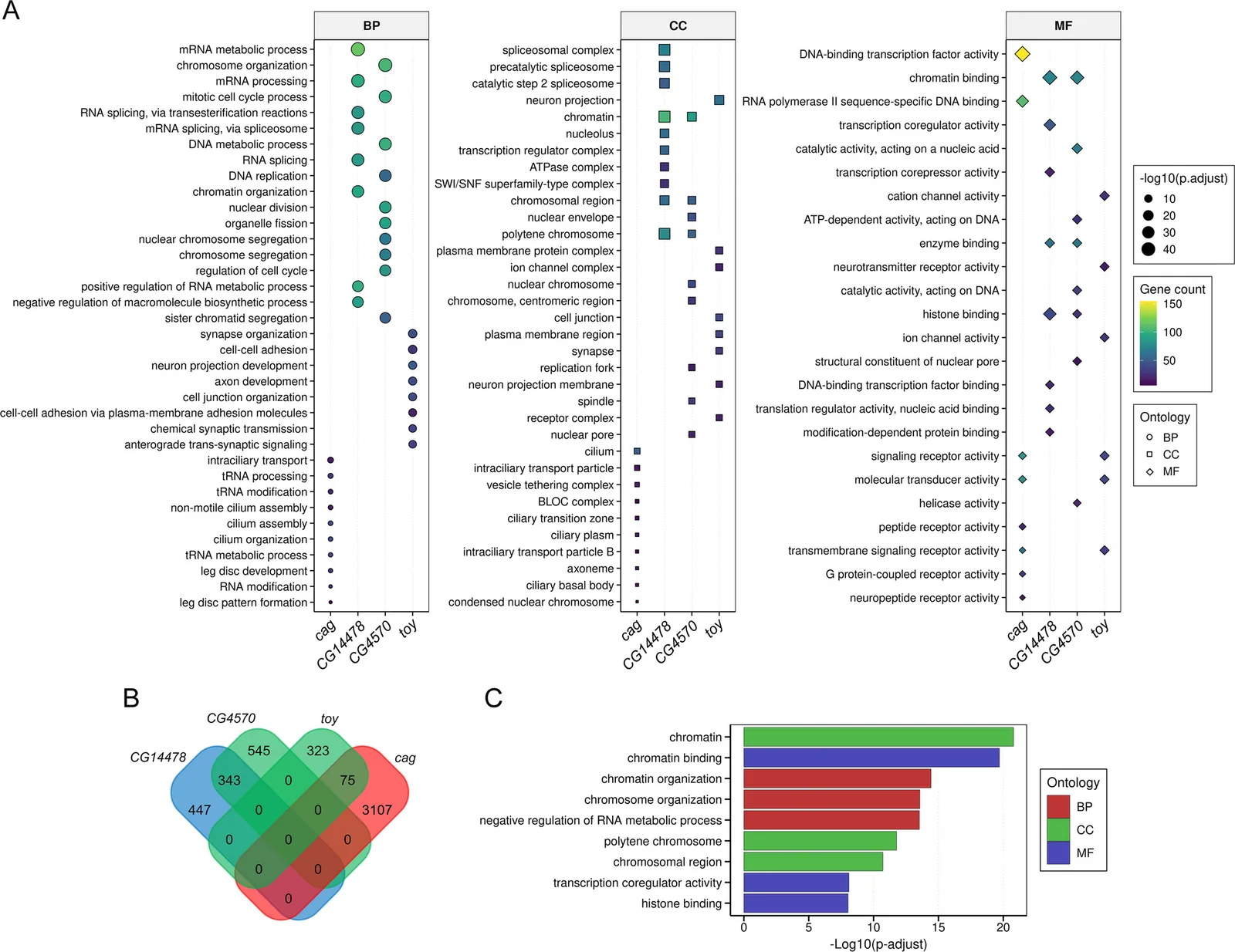
Gene Set Enrichment Analysis (GSEA) for the gene co-expression network of *CG14478*, *CG4570*, *cag*, and *toy*. **A** GSEA was performed against the Gene Ontology (GO) database, encompassing the Biological Process (BP), Cellular Component (CC), and Molecular Function (MF) ontologies. Statistically significant GO terms were defined by an enrichment *p* value (adjusted) threshold of < 0.05. **B** Venn diagram of co-expressed genes for *CG14478*, *CG4570*, *cag*, and *toy*. **C** Top GO terms of shared co-expressed genes between *CG14478* and *CG4570*

The co-expression network of *CG14478*, a gene that retains the conserved catalytic DDE domain, was significantly enriched in terms related to RNA metabolism and chromatin organization (Fig. 5A). Biological processes included mRNA processing, RNA splicing, and chromatin organization. Enriched molecular function terms included chromatin binding, transcription factor binding, and coregulator activity, while enriched cellular components comprised spliceosomal complexes, transcription regulator complexes, chromatin, and the nucleolus (Fig. 5A).

In contrast, the network of *CG4570*, which retained only the ancestral HTH DNA-binding domain, was strongly associated with chromosome and cell cycle functions (Fig. 5A). Enriched terms included chromosome organization, mitotic cell cycle processes, and DNA replication (biological process); chromatin and histone binding (molecular function); and chromatin, nuclear envelope, and replication fork (cellular component) (Fig. 5A).

Notably, 343 genes were co-expressed in both the *CG14478* and *CG4570* networks (Fig. 5B). GO enrichment of these shared genes revealed significant overrepresentation of chromatin-related terms, including chromatin organization (biological process), chromatin binding (molecular function), and chromatin (cellular component) (Fig. 5C).

For the previously characterized genes, *cag* (a *pogo*-derived gene) showed enrichment for ciliary and nuclear functions, including intraciliary transport, cilium assembly, tRNA processing, and chromatin binding (Fig. 5). This supports its known role in centromere function (via its CENP-B domain) and suggests additional, possibly ancient, roles in cellular organization [25, 50, 61]. Gene *toy* (a PAX family progenitor) networks were enriched for neuronal development and signaling, including synapse organization, neuron projection development, cation channel activity, and neurotransmitter receptor activity (Fig. 5), consistent with its established role as a transcriptional regulator in neurogenesis and eye development [49, 62, 63].

To more robustly position the domesticated genes within developmental regulatory networks, we performed Weighted Gene Co-expression Network Analysis (WGCNA) [64]. All four analyzed genes were assigned to distinct modules. Consistent with their expression profiles, the eigengenes of the modules containing *CG14478* and *CG4570* exhibited high connectivity to early embryogenesis (0–8 h), and the *CG14478* module was additionally associated with ovarian tissue (Additional file 7: Fig. S6A). Neither gene functioned as a network hub; however, *CG14478* ranked at the 70th percentile of intramodular connectivity, exceeding *CG4570*, which ranked at the 50th percentile (Additional file 7: Fig. S6B). Although the two genes belong to different modules, the *CG14478* and *CG4570* modules are highly interconnected within the global network (Additional file 7: Fig. S6C). This observation corroborates our earlier finding of overlapping co-expressed genes and suggests that these independently domesticated proteins have become integrated into physically linked and potentially coordinated pathways, particularly those associated with chromatin biology.

Collectively, these findings suggest that both *CG14478* and *CG4570* have been integrated into essential host functions, with distinct yet potentially coordinated roles. *CG14478* is predominantly expressed in ovarian tissue and is associated with RNA and chromatin regulation, likely functions in nucleic acid metabolism. In contrast, *CG4570* shows peak expression during early embryogenesis, suggesting a specialized role in cell cycle and chromatin dynamics through its DNA-binding activity. Their shared co-expressed genes and complementary expression patterns suggest that despite their distinct domain origins, both genes have been integrated into overlapping regulatory pathways centered on chromatin biology and transcriptional control. These predictions for *CG14478* and *CG4570*, along with corroborating data for the established genes *cag* and *toy*, provide a functional context for their evolutionary domestication and a basis for future experimental validation.

## Discussion

### *Tc1/mariner*-derived genes: rare but impactful domestication events

TEs are increasingly recognized as important sources of genomic innovation, capable of evolving from parasitic sequences into functional host genes through molecular domestication. Despite the widespread presence of *Tc1/mariner* elements across eukaryotes, successful domestication events are relatively rare, requiring a series of unlikely steps: neutral fixation of a TPase copy, loss of mobility, functional repurposing, and subsequent refinement under selective constraints. The sparse phylogenetic distribution coupled with the abundance of decaying pseudogenes and annotation artifacts underscores that only a minority of *Tc1/mariner*-derived sequences evolve into stable, functional host genes [5, 31, 32].

Through a systematic genomic survey of 43 *Drosophila* species, we identified five genes unequivocally domesticated from the *Tc1/mariner* superfamily. These genes include four highly conserved genes (*CG14478*, *CG4570*, *cag*, and *toy*) and one lineage-restricted gene, *LOC26532114*, found only in the *D. obscura* species group. Each fulfills stringent criteria for molecular domestication: loss of transposition-associated features (TIRs, TSDs), retention under purifying selection, conserved microsynteny, and detectable expression.

Beyond *Drosophila*, *Tc1/mariner* domestication process has yielded notable examples across eukaryotes. In addition to the *cag* and *toy* genes described previously and re-evaluated in this study [23, 25], *SETMAR* provides another definitive example of *mariner* TPase domestication [19, 20]. In this hominid-specific fusion gene, an *Hsmar1*-derived TPase domain, partnered with a histone methyltransferase SET, has been co-opted for functions in DNA repair [65, 66]. Despite this recurring theme, domesticated genes remain a minority among the vast reservoir of TE-derived sequences—a pattern our findings reinforce within the *Drosophila* genus.

### Dual pathways of *Tc1/mariner* module co-option in the evolution of *CG14478* and *CG4570* genes

The identification of *CG14478* and *CG4570* in *Drosophila* provides further evidence suggesting that the *Tc1/marin*er superfamily is a recurrent source of new host genes. The case of *CG14478*, which retains an intact DDE catalytic domain while its HTH domain has diverged, parallels key examples of “catalytic module” co-option. The most famous analog is the RAG1 recombinase in vertebrates, derived from a *Transib* TPase, where the conserved DDE domain was repurposed for V(D)J recombination while its DNA-binding specificity was fundamentally altered by the host [67, 68]. Similarly, the mammalian *PGBD5* gene, derived from a *piggyBac* TPase, retains its catalytic residues and has been implicated in site-specific DNA rearrangements in the course of neural development [69, 70]. Like *CG14478*, these genes maintain the ancestral endonuclease scaffold but have acquired novel regulatory partners and expression contexts.

The canonical DDE catalytic triad of CG14478 maintains the ancestral catalytic fold and metal-coordinating geometry, supporting its conserved endonucleolytic function. However, a subtle yet notable structural divergence is observed: a one-residue insertion between the D2 and E3 residues distinguishes the CG14478 triad from its ancestral TPase counterpart. Such micro-insertions likely arise through replication slippage or localized duplication during domestication and represent a molecular signature of functional adaptation in domesticated nucleases, as observed in other systems (e.g., RAG1) [67]. Additionally, the side chain of D413 (D2) adopts a ~ 180° rotation relative to ancestral Mariner-4 and Mos1 TPases. Such conformational variability is not unprecedented in DDE/D enzymes. Studies on retroviral integrases and TPases have shown that catalytic aspartates can adopt alternative rotameric states without abolishing activity, particularly if metal ion coordination is maintained by other residues or water molecules [13, 71, 72]. This flexibility may relate to functional adaptations in substrate recognition or regulation [73–76]. In CG14478, D413’s conserved backbone and surrounding pocket suggest metal coordination remains possible, indicating the reorientation may reflect functional specialization. Its co-expression with RNA-processing factors and ovarian enrichment further suggest a role in germline genome integrity or transcriptional regulation.

A notable and potentially key feature of CG14478 is the predicted presence of an IDR, replacing the structured ancestral HTH domain. This evolutionary replacement of a defined DNA-binding motif with a disordered region is a significant transformation which suggests a shift from high-affinity, specific binding to a more flexible, regulatory role. IDRs are known to facilitate dynamic, multivalent interactions with diverse partners such as nucleic acids, proteins, and ribonucleoprotein complexes [77–79].

We speculate that the IDR in CG14478 serves multiple, non-exclusive functions. First, it may act as a flexible tether, allowing its catalytic domain to scan genomic contexts, similar to disordered regions in Transcription Factor II D (TFIID) [80, 81]. Second, it could function as a dynamic interaction hub, recruiting partners for RNA or chromatin regulation, analogous to the integrative platform in p53 [82, 83]. Third, the IDR may facilitate liquid–liquid phase separation, concentrating CG14478 into functional biomolecular condensates [84, 85]. Finally, fourth, it may serve as a central regulatory module, where post-translational modifications like phosphorylation control its activity, comparable to regulation described in FET family proteins [86, 87].

*CG4570* illustrates the “DNA-binding module” domestication pathway, where the TPase catalytic domain is lost and the HTH domain is repurposed for specialized DNA binding. This parallels the origin of CENP-B from a *pogo* TPase, which uses its HTH domain to bind centromeric DNA without transposition activity [24, 25]. Similarly, in plants, transcription factors like FHY3/FAR1, derived from *Mutator*-like TPases, function via retained DNA-binding domains to regulate photomorphogenesis [88, 89]. *CG4570* fits this model: its truncated structure, strong purifying selection, embryonic expression peak, and co-expression with chromatin organization genes suggest a role as a sequence-specific DNA binder involved in chromatin organization or transcriptional regulation during early development.

The simultaneous presence of both domestication modes (retained catalysis in CG14478 and retained DNA-binding in CG4570) within the same TE family in a single genome underscores the evolutionary versatility of transposons. These findings illustrate the versatility of TE domestication, demonstrating that host genomes can independently co-opt and repurpose either the catalytic domain or the DNA-binding domain, each pathway fulfilling a unique adaptive niche. Although *CG14478* and *CG4570* originated in different domains, they show complementary expression and share many co-expressed partners, particularly in chromatin-related functions. This indicates that they have been integrated into overlapping regulatory networks focused on chromatin biology and transcriptional control, demonstrating how domestication can converge distinct TE-derived genes into coordinated cellular roles. Furthermore, the strong purifying selection acting on both genes (dN/dS ~ 0.1) indicates that they have assumed essential, non-redundant cellular functions, a hallmark of deeply domesticated genes like *SETMAR* in primates [18].

We note that the co-option of both catalytic and DNA-binding modules within a single domesticated *Tc1/mariner* derivative remains theoretically possible but has not been observed. Even *SETMAR*, which might appear to be such a case, has lost the catalytic activity of its transposase domain and relies solely on its DNA-binding function. This empirical pattern suggests that once freed from the constraints of transposition, these domains tend to evolve toward functional specialization, either catalysis or binding, rather than maintaining both ancestral activities.

### Validated benchmarks of *cag* and *toy* gene domestication from the *Tc1/mariner* transposons

The re-evaluation of the well-characterized domesticated genes *cag* and *toy* served as a methodological and evolutionary benchmark. Applying our systematic domestication criteria to these known examples validated our analytical framework and provided a conserved functional baseline against which the newly identified genes *CG14478* and *CG4570* could be compared and contextualized.

Based on its expression profile and co-expression network, we suggest that the *pogo*-derived *cag* gene functions in ciliogenesis, with a primary role in spermatogenesis. The significant enrichment of intraciliary transport and cilium assembly pathways, coupled with peak *cag* expression in testes, suggests a role in sperm flagellum formation, a process directly dependent on the centriole-derived basal body. This aligns with the established pattern of TPase co-option for centriolar and ciliary functions [25].

Furthermore, the concurrent enrichment of tRNA processing and RNA modification pathways among *cag* co-expressed genes raises the possibility that Cag protein may also facilitate the precise translational control required to meet the high protein synthesis demands of spermatogenesis and embryogenesis. This would position *cag* as a factor potentially linking ciliary assembly to localized RNA metabolism [90].

The *Tc1/mariner*-derived gene *toy* is suggested to act as a transcriptional regulator in *D. melanogaster* neuronal development and synaptic signaling [23, 49]. Its expression is low and widespread, but absence in early embryos and aged females indicates a stage- and tissue-specific functions in neural maturation or maintenance. Functional enrichment supports its role as a progenitor of the PAX family, key regulators of neurogenesis and eye development [91–93]. Its conserved HTH domain, derived from an ancestral TPase DNA-binding module, may allow *toy* to regulate neural and synaptic genes, analogous to domesticated TPase-derived factors like vertebrate PAX6 [94].

### A snapshot of recent, lineage-specific domestication

Our analysis shows that domestication events vary across lineages. While certain genes like *cag* and *toy* are widely conserved, others, such as *CG14478* and *CG4570*, are broadly present in invertebrates and some vertebrates but were lost in amniotes like birds and mammals, likely due to redundancy or maladaptation. However, the presence of *CG14478*- and *CG4570*-like genes across diverse phyla, including vertebrates, likely reflects multiple independent domestication events of *Tc1/mariner* TPases, a phenomenon known as recurrent evolution [24, 27, 40], rather than a single ancestral domestication at the base of these lineages.

*LOC26532114* exhibits features suggestive of a recent, lineage-specific domestication event in the *D. obscura* species group. Its limited distribution suggests a single domestication event in the clade’s ancestor, without further spread, possibly due to a lack of adaptive relevance or recent loss. The gene also displays partial domain retention and decay.

While *LOC26532114* retains strong overall sequence similarity to ancestral *Tc1/mariner* TPases, domain analysis indicates the complete loss of the canonical DDE catalytic triad, with only a fragment of the IS630 TPase domain preserved alongside an intact N-terminal HTH domain. Unlike other strongly conserved domesticated genes, it shows mild selective constraint, typical of early domestication, where the function has not yet been fixed or become essential [26, 95–98].

Further evidence of recent regulatory integration comes from its gene structure and promoter association. The acquisition of a conserved intron in the 5′ UTR, absent in the ancestral transposon, represents a clear signature of host-genome assimilation. Moreover, the extremely close proximity of its TSS to that of the upstream gene strongly suggests promoter sharing or hijacking. This arrangement indicates that *LOC26532114* likely achieved expression by co-opting the regulatory machinery of a neighboring gene, a mechanism observed in other domesticated TEs that integrated near host regulatory elements [97, 99, 100]. For the remaining ancient domesticated genes, promoter architecture varies. Gene *cag* shares a bidirectional promoter with the adjacent gene *CG12942*, and their expression remains significantly correlated. This probably indicates a regulatory partnership maintained over deep evolutionary time. In contrast, *CG14478*, *CG4570*, and *toy* show no evidence of promoter sharing with neighboring genes. This likely reflects the extensive evolutionary interval since their initial domestication. Over tens to hundreds of millions of years, any ancestral TE-derived promoter sequences would have been eroded and replaced by canonical host regulatory elements. Consistent with this view, analysis of available modENCODE data (ChIP-seq, CAGE, RNA Pol II occupancy) in *D. melanogaster* confirms that *CG14478*, *CG4570*, and *toy* are transcribed from standard host core promoters (data not shown). Together, these observations span a continuum of regulatory integration: from the recent, visible promoter-hijacking of *LOC26532114*, through the ancient yet still detectable partnership at *cag*, to the fully assimilated host promoters of *CG14478*, *CG4570*, and *toy* where the original mechanism has been obscured by deep evolutionary time.

Compared to well-established domesticated genes, *LOC26532114* lacks the broad expression pattern and strong functional enrichment seen in genes like *CG14478* or *toy*. It may encode a protein with a minor, lineage-specific role, or it may be on an evolutionary trajectory toward pseudogenization if it does not confer a significant selective advantage.

In the broader context of TE domestication, *LOC26532114* exemplifies the dynamic and ongoing nature of host-transposon molecular coevolution. It underscores that domestication is not a binary switch but a continuum—from recently captured, slightly functionalized elements to deeply integrated, essential host genes. Similar cases of lineage-specific domestication have been reported for *pogo*-derived genes in certain mammalian clades, highlighting that such “young” domestication events are a recurring feature of genome evolution [26].

## Conclusions

This study establishes a stringent framework for distinguishing high-confidence molecular domestication of transposons from genomic debris. It delineates two distinct pathways of *Tc1/mariner* module co-option and illustrates how domestication unfolds as a progressive evolutionary trajectory, from newly established lineage-specific genes to deeply integrated, ancient host factors. While this approach is robust for identifying deeply conserved events, it may be less sensitive to younger, lineage-specific domestications, underscoring the importance of complementary methods to fully capture the evolutionary continuum of host-transposon co-option.

While our bioinformatic approach provides in silico evidence for the domestication and functional specialization of *CG14478* and *CG4570*, these findings necessitate experimental confirmation. Next steps should include experimental validation of newly identified candidates and investigation of how structural features, like intrinsic disorder, contribute to functional innovation. Specific, testable hypotheses emerging from this work are as follows: (1) *CG14478* retains a DDE endonuclease fold and may operate in RNA or chromatin-related processes; (2) *CG4570* maintains only the DNA-binding domain and is likely involved in chromatin or cell-cycle regulation; (3) *LOC26532114* represents a recent domestication event and could serve as a model for studying regulatory integration. These predictions are supported by phylogenomic, structural, and co-expression evidence. Future experimental work, including knockout, DNA-binding, and interaction studies, can validate these hypotheses. By framing TE domestication as a dynamic and predictable process, this work offers a generalizable roadmap for exploring how genomic conflict drives functional innovation across eukaryotes.

While our findings delineate two predominant pathways of *Tc1/mariner* module co-option in *Drosophila*, we acknowledge that other evolutionary outcomes are possible. The retention of both functional modules, or the fusion of transposase domains with novel host domains beyond what is seen in SETMAR or PAX proteins, may yet be discovered as more genomes are analyzed.

## Methods

### Identification of *Tc1/mariner*-derived genes

Genomes of 43 Drosophilinae subfamily species (42 *Drosophila* and one *Scaptodrosophila*) and their annotations were downloaded from NCBI. All data sources are listed in Additional file 8: Table S1. The TE protein dataset was derived from the Repeat Protein Database (RepeatPeps) implemented in the RepeatMasker package [101]. To ensure homology to characterized TE families, sequences annotated with “UN-,” “Unknown,” or “Other” prefixes were excluded, as these classifications indicate a lack of significant homology to known TE families. Furthermore, sequences identified as containing premature termination codons (PTCs) based on FASTA header annotations were removed. Only sequences originating from the RepBase and Dfam repositories were retained for downstream analysis [102, 103].

Protein domains were annotated using the Conserved Domain Database (CDD) fetched from the NCBI FTP server [44]. A local rpsblast + search [104] was executed with the following parameters: *rpsblast* + *-db CDD.file -query repeatpeps_filtered.fasta -evalue 0.05 -num_threads 64 -outfmt 5*. The resulting XML output was converted to tabular format using the blastxml_to_tabular.py script from the Galaxy toolkit [105]. The tabular domain data were imported into the R environment [106].

Candidate TE-derived genes were identified via tblastn [104] homology searches using a curated library of TE-encoded proteins of *Tc1/mariner* superfamily as query sequences against annotated coding sequences (CDS) derived from *Drosophila* species. Parallel computation was implemented via GNU Parallel to accelerate pairwise alignment [107]. Initial alignments were subjected to a bitscore-based filtering threshold, retaining only those alignments exceeding the maximum bitscore obtained from control alignments against randomly generated CDS sequences.

To assess copy number, candidate genes exhibiting significant homology to TE proteins and lacking introns (i.e., non-spliced) were aligned to their respective reference genomes under an end-to-end alignment paradigm. This was performed using minimap2 under the “asm10” pre-set, optimized for mapping divergent sequences [108]. Alignments covering ≥ 90% of the query gene length were retained, and copy number was quantified based on the count of distinct genomic loci fulfilling these alignment criteria.

To confirm the absence of TIRs and TSDs, we extracted 500 bp sequences upstream and downstream of each candidate gene. These flanking sequences were analyzed for homology using blastn. Candidates showing no significant TIR-like structures (*e* value > 0.05) were retained, supporting their classification as non-autonomous, gene-derived sequences.

### Orthology assignment, gene structure, and microsynteny analysis

Orthologous relationships were determined by querying the OrthoDB v12.2 database [33] via its REST API (access date 13.09.2025), using NCBI gene identifiers and restricting the search to eukaryotic orthology groups. For local synteny assessment, the GENESPACE tool [109] was applied in conjunction with ortholog predictions from the DrosOMA database [110]. For the *Drosophila teissieri* gene *LOC122624997*, for which no orthologs were detected via DrosOMA, microsynteny analysis was conducted through manual curation of the flanking genes in the annotated genomes of related species possessing orthologous sequences.

The domain architecture of TE element-derived genes was characterized using the CDD [44], with all predictions subjected to subsequent manual curation.

Exon–intron structure of studied genes was visualized using WormWeb exon–intron graphic maker (http://wormweb.org/exonintron).

Translation initiation sites were predicted using TISpredictor [53].

### Sequence analysis and phylogenetic inference

For each domesticated gene, we attempted to identify *Tc1/mariner* TPase sequences directly in the genomes of the species included in the ortholog alignments. However, the extensive sequence divergence and degradation of ancestral transposon copies in these species precluded retrieval of full-length TPases with sufficient homology to serve as reliable phylogenetic markers. As an alternative, we searched for intact *Tc1/mariner* TPases in the genomes of the most closely related drosophilid species available. These sequences were retrieved from the RepeatPeps database based on highest bitscore rankings against the respective domesticated protein. Lepidopteran and non-*Drosophila* dipteran species (*Bombylius major*, *Bombyx mori*, *Bombyx mandarina*, *Bombylius discolor*, *Anopheles gambiae*) were included as outgroups. Multiple sequence alignment was performed using MAFFT [111] with the “*–auto*” parameter. Alignments were subsequently inspected manually. To improve phylogenetic signal, poorly conserved regions and gaps were removed using trimAl [112] in its “maximum likelihood” mode.

Phylogenetic trees were reconstructed using the maximum likelihood method as implemented in the phangorn R package [113]. The best-fitting substitution model, JTT+G(4)+I, was selected based on the modelTest function. Branch support was assessed using 100 bootstrap replicates. Final tree visualization and annotation were performed using the interactive Tree of Life (iTOL) web platform [114].

### Testing for purifying selection

To assess the presence of purifying selection on amino acid sequences derived from TE genes, we calculated the non-synonymous-to-synonymous substitution rate ratio (dN/dS) using codon-based models. Phylogenetic trees were reconstructed following the same methodology employed in earlier steps, utilizing only *Drosophila* protein-coding sequences. Corresponding DNA alignments were realigned on a codon-basis with PAL2NAL [115]. Subsequently, the PAML software was applied to estimate site-specific dN/dS ratios [116]. For each gene alignment, dN/dS values were estimated under the M0, M1a, and M2a evolutionary models. Likelihood ratio tests were subsequently conducted to compare model fits, specifically evaluating M0 against M1a and M1a against M2a.

#### DDE motif analysis

To characterize the conserved DDE catalytic triad in *Tc1/mariner*-derived gene, multiple sequence alignments were generated using MAFFT [111]. Putative DDE motifs were identified via manual inspection of conserved alignment positions.

For structural analysis, the three-dimensional model of CG14478 was extracted from the AlphaFold Protein Structure Database (AF-Q6NR30-F1) [117]. The experimentally resolved crystal structure of the Mos1 TPase catalytic domain from *Drosophila mauritiana* (PDB: 2F7T) [118] was obtained from the Protein Data Bank [119]. Catalytic aspartate (D) and glutamate (E) residues were mapped onto each structure based on the corresponding multiple sequence alignment. Structural superposition was performed in PyMOL using the “super” command, followed by visualization within the same environment [59].

A tertiary structure model of the *Drosophila bipectinata Mariner-4_DBp_tp* TPase was predicted using AlphaFold2 with default parameters [57] and structurally aligned with the CG14478 model. DDE residue positions were annotated manually in both superimposed structures. Alignment quality was assessed using the root-mean-square deviation (RMSD, in Å) and the alignment score returned by PyMOL.

Identification of intrinsically disordered protein regions (IDRs) was performed using AIUPred software [58].

### Reconstruction and analysis of gene co-expression networks

Gene co-expression networks were reconstructed and analyzed using modENCODE expression data for *Drosophila melanogaster* [48]. Both tissue-specific (“Tissue”) and developmental time-course (“Development”) expression datasets were employed. This analysis was restricted to the four domesticated genes with clear orthologs in *D. melanogaster* (*CG14478*, *CG4570*, *cag*, and *toy*), as the modENCODE expression datasets are specific to this species. The lineage-specific gene *LOC26532114* from the *D. obscura* group was excluded from this particular analysis.

Gene-level co-expression analysis used published RPKM values from FlyBase [120]. Data were processed in R, and pairs of genes with a Spearman correlation coefficient > 0.7 (*p* < 0.05) were defined as co-expressed. Only gene pairs showing significant co-expression in both the “Tissue” and “Development” datasets were retained for network reconstruction.

In addition to the Spearman correlation, Weighted Gene Co-expression Network Analysis (WGCNA) was performed using the WGCNA R package on the same expression dataset [64]. Gene correlation matrices were transformed into an adjacency matrix using a soft power threshold of 4. Modules were identified using a minimum cluster size of 30 and a deep split parameter of 2, with each module assigned a unique color identifier. Genes were classified as “hub” (> 90th percentile) or “peripheral” (0–90th percentile) based on their within-module connectivity (kWithin). Module eigengenes were calculated, and a module-module interaction network was constructed from adjacency matrix values using a Spearman correlation cutoff of 0.4. Simple Gene Set Enrichment Analysis (GSEA) was performed on each module using Gene Ontology (GO) biological process terms. Only GSEA terms shared between modules of interest and their first-shell neighboring modules are shown. Network visualization was performed using the ggnetwork R package.

Functional enrichment analysis was performed on co-expressed gene sets using the enrichGO function from the clusterProfiler package [121], assessing overrepresentation in GO categories for Biological Process, Cellular Component, and Molecular Function. Redundant GO terms were reduced using the “*simplify*” function within the same package, followed by manual curation of the resulting enriched terms.

### Gene expression and ChIP-seq analyses in non-*D. melanogaster* species

To characterize the expression profiles of genes lacking orthologs in *D. melanogaster*, we analyzed publicly available RNA-seq data from 12 *Drosophila* species. These data were retrieved from the Short Read Archive (SRA) using the edirect (https://github.com/ncbi/docker/tree/master/edirect) and fasterq-dump utilities (https://github.com/ncbi/sra-tools) (dataset details are provided in Supplemental Table S1). Raw reads from each sample were trimmed with Trim Galore (https://github.com/FelixKrueger/TrimGalore) and then aligned to the corresponding genome using HISAT2 [122]. Alignments were processed with SAMtools [123]. Gene-level coverage was extracted on a per-position basis via samtools depth and normalized to reads per kilobase per million mapped reads (RPKM).

Raw data of genome binding/occupancy (ChIP-seq) of RNA polymerase II in ovarian tissue of *Drosophila pseudoobscura* was obtained from the Gene Expression Omnibus (GEO) database (GSE213379). Trimmed sequenced reads were aligned to the *D. pseudoobscura* genome with Bowtie2. Aligned reads were sorted by coordinates, filtered of most multi-mapped reads (*samtools view -Sbh -q 10*) and cleaned of duplicates (samtools rmdup) using SAMtools [123]. Aligned files in BAM format were converted to BigWig format with a bin size of 10 nt and normalized using the RPGC method (number of reads per bin/scaling factor for 1 × average coverage of the genome) using the bamCoverage script (*bamCoverage –binSize 10 –normalizeUsing RPGC –effectiveGenomeSize (total length of the genome) –extendReads (for pair-end data)*) implemented in the deepTools2 package [124]. Next, input sample values were subtracted from treatment sample values using the bigwigCompare script. Enrichment was visualized in the Integrative Genome Viewer (IGV) [125].

## Supplementary Information

Additional file 1: File 1 Best alignments (tblastn) of studied genes to Repeat Protein Database (RepeatPeps).

Additional file 2: Fig. S1 Molecular domestication of a *cag* gene derived from a *Tc1/mariner* TPase in *Drosophila*. (A) Pairwise alignment (gray) between the domesticated protein (bottom) and its ancestral TPase (top), identified by tblastn. Domains are annotated according to PFAM. CENP-B_n—CENP-B N-terminal DNA-binding domain; CENPB—putative DNA-binding domain in centromere protein B; HTH—helix-turn-helix TPase DNA-binding domain; DDE—DDE superfamily endonuclease. A histogram displays amino acid conservation across the alignment (score: 11 = identical, 10 = conserved properties). The dN/dS ratio for orthologs of the domesticated gene is shown at right. The reported dN/dS ratio is statistically supported by a likelihood ratio test (*p* < 0.05). (B) Maximum likelihood phylogeny of the domesticated orthologs and a selection of ancestral *Tc1/mariner* superfamily TPases. The orthologs of *cag* from *Bombylius discolor*, *Bombyx mori*, and *Bombyx mandarina* were used as an outgroup. Bootstrap values after 100 replicates are indicated. (C) Genomic microsynteny of the locus containing the domesticated gene across *Drosophila* species. The orthologous region from *D. melanogaster*, including the query gene and its flanking sequences, is uniformly highlighted in a distinct color in all species. Faded coloring denotes non-orthologous genes. Arrows denote transcriptional direction. (D) Normalized expression (RPKM) of *cag* in developmental stage- and tissue-specific RNA-seq datasets.

Additional file 3: Fig. S2 Molecular domestication of a *toy* gene derived from a *Tc1/mariner* TPase in drosophilid species. (A) Pairwise alignment (gray) between the domesticated protein (bottom) and its ancestral TPase (top), identified by tblastn. Domains are annotated according to PFAM. HTH—helix-turn-helix TPase DNA-binding domain; DDE—DDE superfamily endonuclease; PAX—paired-box domain. A histogram shows amino acid conservation across the alignment (score: 11 = identical, 10 = conserved properties). The dN/dS ratio for orthologs of the domesticated gene is displayed at the right. (B) Maximum likelihood phylogeny of the domesticated orthologs and a selection of ancestral *Tc1/mariner* superfamily TPases. The orthologs of *toy* from *Bombylius discolor*, *Bombylius major*, *Bombyx mandarina*, and *Anopheles gambiae* were used as an outgroup. Bootstrap values after 100 replicates are shown. (C) Genomic microsynteny of the locus containing the domesticated gene across *Drosophila* species. The orthologous region from *D. melanogaster*, including the query gene and its flanking sequences, is uniformly highlighted in a distinct color in all species. Faded coloring denotes non-orthologous genes. Arrows indicate transcriptional direction. (D) Normalized expression (RPKM) of *toy* in developmental stage- and tissue-specific RNA-seq datasets.

Additional file 4: Fig. S3 Prediction of translation initiation sites in *D. obscura LOC111072153*. The values indicate the Kozak similarity score predicted by the machine learning model.

Additional file 5: Fig. S4 Features distinguishing decaying *Tc1/mariner*-derived pseudogenes from domesticated genes. (A) Genomic microsynteny of the locus containing the domesticated gene across *Drosophila* species. The orthologous region from *D. teissieri*, including the query gene and its flanking sequences, is uniformly highlighted in a distinct color. A lighter color signifies the absence of an ortholog. (B) Comparative gene organization of *LOC26532114* and its putative orthologs. White and black regions on the scheme denote untranslated and coding regions, respectively. (C) Maximum likelihood phylogeny of the domesticated orthologs and a selection of ancestral *Tc1/mariner* superfamily TPases. Bootstrap values after 100 replicates are shown. (D) RPKM-normalized expression and sequencing coverage profiles across gene bodies..

Additional file 6: Fig. S5 AIUPred analysis of the CG14478 (UniProt: Q6NR30). Prediction profile is generated using the default options (AIUPred—only disorder, default smoothing). A score between 0 and 1 for each residue, corresponding to the probability of the given residue being part of a disordered region. Annotated regions from PFAM.

Additional file 7: Fig. S6 WGCNA analysis of *Tc1/mariner*-derived genes in *D. melanogaster* gene expression. (A) Heatmap of module eigengene values for the modules containing *cag* (brown), *toy* (red), *CG14478* (black), and *CG4570* (turquoise) across modENCODE samples. Tiles are marked with single (0.2–0.3), double (0.3–0.4), or triple (> 0.4) asterisks according to eigengene value ranges. (B) Boxplot of within-module connectivity (kWithin) distributions for the four modules. The kWithin values for *cag*, *toy*, *CG14478*, and *CG4570* are shown as gray dots within their respective modules. (C) Module-module interaction network constructed using a Spearman correlation cutoff of 0.4. Modules containing *Tc1/mariner*-derived genes are indicated by ellipses with sticks, with relevant GSEA terms displayed.

Additional file 8: Table S1 Data sources.

## Data Availability

All genomic data used in this study are publicly available from NCBI as listed in Additional file 8: Table S1.

## Abbreviations

BLAST: Basic Local Alignment Search Tool
CAGE: Cap Analysis Gene Expression
CDD: Conserved Domain Database
CDS: Coding sequence
CENP-B: Centromere protein B
ChIP-seq: Chromatin immunoprecipitation sequencing
CNS: Central nervous system
DDE/D: Aspartate-Aspartate-Glutamate/Aspartate (catalytic motif)
dN/dS: Non-synonymous to synonymous substitution rate ratio
DrosOMA: *Drosophila* Orthology Matrix
FET: Fused in Ewing sarcoma/Ewing sarcoma breakpoint region 1/TATA-binding protein-associated factor 15 (protein family)
FHY3/FAR1: Far-red elongated hypocotyl 3/Far-red impaired response 1 (plant transcription factors)
GEO: Gene Expression Omnibus
GO: Gene Ontology
GSEA: Gene Set Enrichment Analysis
HTH: Helix-turn-helix
IDR: Intrinsically disordered region
IGV: Integrative Genomics Viewer
iTOL: Interactive Tree of Life
LRT: Likelihood ratio test
MAFFT: Multiple Alignment using Fast Fourier Transform
modENCODE: Model Organism Encyclopedia of DNA Elements
NCBI: National Center for Biotechnology Information
ORF: Open reading frame
PAI/RED motifs: Paired box sequences (PAI and RED) in DNA-binding domains
PAML: Phylogenetic Analysis by Maximum Likelihood
PAX: Paired box gene family
PDB: Protein Data Bank
PGBD5: PiggyBac transposable element derived 5
pLDDT: Predicted local distance difference test
PTC: Premature termination codon
RAG1: Recombination activating gene 1
RepeatPeps: Repeat Protein Database
RMSD: Root-mean-square deviation
RNA-seq: RNA sequencing
RPKM: Reads per kilobase per million mapped reads
RPS-BLAST: Reverse Position-Specific BLAST
SETMAR: SET domain and mariner transposase fusion gene
SRA: Short Read Archive
TE: Transposable element
TFIID: Transcription Factor II D
TIGD: Tigger transposable element derived gene family
TIR: Terminal inverted repeat
TPase: Transposase
TSD: Target site duplication
TSS: Transcriptional start site
UTR: Untranslated region
WGCNA: Weighted Gene Co-expression Network Analysis
